# Comparison of Vertical Marginal Discrepancy in High and Low Translucent Monolithic Zirconia Crowns in Repeated Firing Cycles

**DOI:** 10.1055/s-0044-1801303

**Published:** 2025-03-12

**Authors:** Mehrzad Moazzam, Amirhossein Fathi, Mahsa Ghorbani, Ramin Mosharraf

**Affiliations:** 1 Department of Endodontics, Faculty of Dentistry, Islamic Azad University Isfahan (Khorasgan) Branch, Isfahan, Iran; 2 Dental Materials Research Center, Department of Prosthodontics, School of Dentistry, Isfahan University of Medical Sciences, Isfahan, Iran; 3 Dental Research Center, Mashhad University of Medical Sciences, Mashhad, Iran

**Keywords:** vertical marginal discrepancy, zirconium, dental implants, dental materials, implant abutments

## Abstract

**Objective** Increase in vertical marginal discrepancy (VMD) during repeated firing cycles and its clinical outcomes is a major concern for high and low translucent monolithic zirconia crowns. The purpose of this in vitro study was to evaluate and compare VMD in high and low translucent monolithic zirconia crowns in repeated firing cycles.

**Material and Methods** To perform this study, 10 monolithic zirconia crowns made by computer-aided design and computer-aided manufacturing method were used in two groups of five with high and low translucency, which were designed on Zimmer tissue-level implant abutment. The crowns in each group were randomly numbered from 1 to 5 and each underwent 1, 3, and 5 firing cycles. After completing each mentioned cycle, the VMD was measured at eight predetermined points on abutment by optical microscope and their average was recorded for each sample. Data analysis was done by SPSS 22 software through repeated-measure analysis of variance, paired *t* -test, and *t* -test with a 5% significance threshold.

**Results** A total of 240 measurements were made for the VMD, which, due to the presence of five samples in each translucency group and eight examined points in each sample, was finally summed up to six averages for each translucency group in the mentioned three stages of firing cycles. The averages for the low-translucency group after 1, 3, and 5 firing cycles were 76.86, 85.02, and 90.55 μm, respectively, and for the high-translucency group after 1, 3, and 5 firing cycles were 80.38, 87.33, and 97.78 μm, respectively. The average VMD of the samples regardless of the translucency level after 1, 3 and 5 firing cycles was calculated as 78.62, 86.18, and 94.16 μm, respectively.

**Conclusion** This study found that VMD increased with repeated firing cycles, with no significant difference between high- and low-translucency zirconia crowns. Repeated firings significantly raised VMD, but all values remained within clinically acceptable limits, supporting the suitability of both translucency types for clinical use.

## Introduction

Today, all-ceramic dental crowns have gained popularity among patients and dentists due to their aesthetic appeal and suitable mechanical properties. However, from a clinical perspective, the porcelain layer of crowns is prone to chipping and surface wear, potentially leading to restoration failure. 1 2 3 To address these issues, new materials have been developed that aim to fix the defects of previous-generation ceramics while largely preserving their positive qualities. Zirconia is one of these materials, and knowledge of its properties is essential for clinical practice.

Zirconia, a polycrystalline ceramic without a glassy phase, has superior mechanical properties compared with alumina-based ceramics. 2 It exists in several temperature-dependent crystalline forms. At room temperature, zirconia is found in the monoclinic form, but it transitions to cubic and tetragonal crystalline forms after sintering. When cooling, a shift from cubic to tetragonal and then monoclinic forms results in volume expansions of 2.3 and 4.2%, respectively. This volumetric change can introduce fractures and cracks, especially in the tetragonal phase. 4 The addition of yttria to stabilize the tetragonal phase and reduce microcracking due to phase changes, along with advancements in computer-aided design and computer-aided manufacturing (CAD/CAM) technology, has led to the development of new zirconia restorations. Monolithic zirconia is one of the latest of these, suitable for both anterior and posterior regions.

Monolithic zirconia is fabricated using CAD/CAM from a single zirconium oxide block without any veneering porcelain 2 and offers higher fracture strength and flexural strength compared with traditional porcelain restorations. It is also less prone to chipping and allows for a more conservative tooth preparation, which helps preserve healthy dental tissue. 2 3 5 Recently, high-translucency monolithic zirconia has been introduced, featuring a high purity level and minimal porosity. 2 While high-translucency zirconia provides better aesthetic matching to natural teeth, it has lower flexural strength than low-translucency zirconia. Additionally, techniques to enhance translucency—such as particle size reduction and metal oxide additions—can influence other properties, including marginal fit. 6 7 8 9

Achieving a precise marginal fit is crucial for the success and longevity of restorations, as poor fit can result in plaque accumulation, leading to microleakage and ultimately to pulpal lesions, secondary caries, periodontal disease, and bone loss. 4 10 Moreover, suboptimal fit may compromise fracture resistance and reduce the strength of the restoration. Many studies consider a marginal gap of 50 to 120 μm clinically acceptable, with rigorous studies associating gaps below 100 μm with more favorable outcomes. 11 12 The marginal gap of zirconia copings produced via CAD/CAM has been reported to range between 10 and 160 μm, with most falling under 80 μm. 13 14 15 In all-zirconia crowns, marginal gaps of approximately 11 to 58 μm have been observed. 8 16 Studies indicate that marginal gap size is impacted by material thickness, which varies between high- and low-translucency zirconia. The shrinkage from sintering, also related to material thickness, suggests that marginal gap and its changes over repeated firing cycles may differ between high- and low-translucency types. 7 17 18 19

Repeated firing cycles are commonly performed to improve color, contour, and glazing, but they may affect the marginal fit of zirconia crowns, leading to vertical marginal discrepancies (VMDs). Despite numerous studies, current findings on the effects of firing cycles on marginal gaps are inconsistent, differing in their design, sample sizes, and methodologies. 20 21 22 23 24 25 26 27 Variations in methodologies—such as the use of distinct techniques for measuring marginal gaps (e.g., cross-sectional imaging vs. silicone replica technique) and differences in the number of firing cycles applied—have contributed to inconsistencies in results. Given these inconsistencies and the increasing use of monolithic zirconia—especially the high-translucency type—our study aims to investigate the marginal vertical discrepancy in both high- and low-translucency zirconia crowns during repeated firing cycles. The distinction in translucency classification is critical because it influences aesthetic and mechanical properties. The high-translucency zirconia is designed for improved esthetics, particularly for anterior restorations, while the low-translucency zirconia is suited for posterior restorations where strength is prioritized. This classification was selected to evaluate how varying translucency levels impact marginal fit and VMD after repeated firing cycles.

## Material and Methods

In this *in vitro* study, 10 monolithic zirconia crowns were fabricated, divided into two groups of five: one group with high translucency (B light shade, XT Cera Ltd.) and the other with low translucency (Bleach shade, Katana Ltd.).

The zirconia used for high-translucency crowns, XT Cera, contains approximately 5 mol% yttria-stabilized tetragonal zirconia polycrystals (5Y-TZP). This higher yttria content increases the cubic phase ratio in the zirconia, enhancing translucency by reducing birefringence and scattering of light. In contrast, the low-translucency zirconia, Katana Ltd., contains approximately 3 mol% yttria-stabilized tetragonal zirconia polycrystals (3Y-TZP), which has a higher percentage of the tetragonal phase. This composition results in greater strength but lower translucency due to increased grain boundaries that scatter light.

The crowns were designed on tissue-level implant abutments (4.1 × 10 mm, Zimmer Dental, United States) using the 3Shape software (Dental System, Copenhagen, Denmark). The upper section of the abutment was meticulously milled using a milling machine (Up3D P53 CAD/CAM, Shenzhen Up3D Tech Co Ltd.) to achieve an occlusal convergence of 12%. A radial shoulder finish line of 1 mm and a ledge on the axio-occlusal line angle measuring 3 mm in length and 0.5 mm in depth were incorporated into the design to prevent crown rotation and facilitate ease of movement. By utilizing precise milling techniques and adhering to established design parameters, we minimized variability in abutment preparation, thereby enhancing the reliability of the subsequent fit of the monolithic zirconia crowns.

**Fig. 1:**
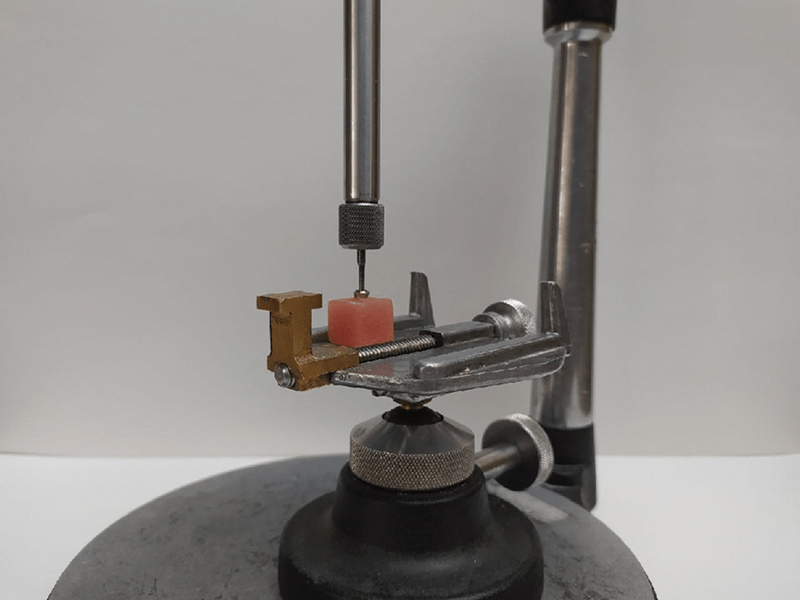
Mounting the abutment in an acrylic resin block using a surveyor.

**Fig. 2:**
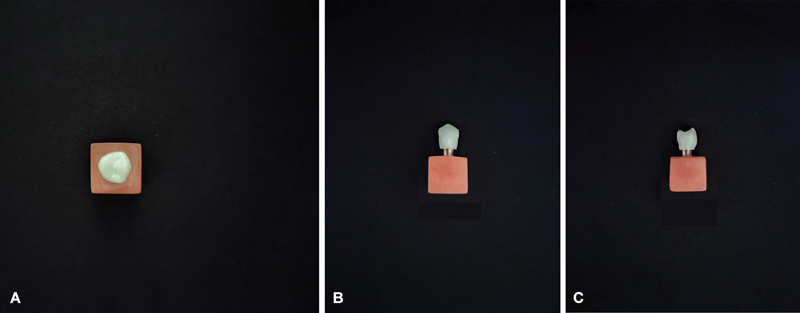
Views of a low-translucency sample: ( **A** ) occlusal, ( **B** ) buccal, and ( **C** ) mesial.

Following abutment preparation, the implant abutment was vertically mounted in self-curing acrylic resin (GC Pattern Resin; GC Corp, Tokyo, Japan) using a surveyor to ensure consistent positioning ( Fig. 1 ). A laboratory scan of the abutment was then created (Scanner Up360 Plus, Shenzhen Up3D Tech Co Ltd.), and the crowns were milled from zirconia blocks using CAD/CAM technology on the same milling machine ( Fig. 2 ), achieving a consistent marginal thickness of 1 mm and a cement space of 30 μm. All manufacturing steps followed the protocols specified by the zirconia manufacturers and were performed by a single technician to ensure uniformity.

The crowns in each group were randomly numbered (1–5) and subjected to firing cycles at intervals of 1, 3, and 5 cycles. The initial firing cycle was completed using a ceramic sintering machine (Auto Sinter 1650, KFP Dental, IRI), which ran for a total of 12.5 hours and held a peak temperature of 1,550°C for 2.5 hours. Subsequent firing cycles were performed in a glazing machine (Programat P300, Ivoclar Vivadent, Liechtenstein) at 930°C with a heating rate of 65°C/min.

Each crown was then placed on the prepared abutment, and VMD was measured after each firing cycle. Measurements were taken at eight predetermined points around the crown margins (mesiobuccal, distobuccal, mesiolingual, and distolingual line angles, as well as midbuccal, midlingual, midmesial, and mid-distal points).

To ensure measurement accuracy and consistency, several precautions were taken. Prior to the experiments, the optical stereomicroscope (SMP 200 Model HP, united States) was calibrated according to the manufacturer's specifications, ensuring precise measurements throughout the study. The digital camera (Moticam 480, Motic Instruments Inc., California, United States) attached to the microscope captured images for analysis at 40× magnification. Each crown was stabilized with a constant force of 5 N applied by a verticulator (Handy Sil; Mestra) to maintain consistency during measurements. VMD measurements were processed using image analysis software (Motic Image Plus 2.0, Motic Instruments Inc.).

To minimize operator variability, all measurements were performed by a single trained technician, who adhered to a standardized protocol for positioning the crowns and capturing images. The technician received thorough training in the use of the equipment and the measurement techniques. Furthermore, the technician performed blinded measurements twice, with a 7-day interval between each session. The consistency of these repeated measurements was evaluated using an intraclass correlation coefficient of 0.95, indicating excellent reliability. Furthermore, all images were analyzed using the same image analysis software, and the analysis procedure was conducted in a consistent manner for each sample, further ensuring reliability in the measurement of VMDs. This study was approved by the Ethics Committee of Isfahan University of Medical Sciences (IR.MUI.REC.1401.025).

This study used a sample size of 10 crowns, aligning with previous *in vitro* studies examining zirconia restorations. 26 To enhance reliability despite the pilot sample, we assessed eight measurement points around each crown (mesiobuccal, distobuccal, mesiolingual, distolingual line angles, midbuccal, midlingual, midmesial, and mid-distal points). This method, which exceeds the typical four-point measurement approach used in prior studies, allowed for a more detailed analysis of marginal discrepancies across the crown surface.

All collected data were recorded and analyzed using statistical software (IBM SPSS Statistics for Windows, v22.0; IBM Corp). Normality of the data was assessed using the Shapiro–Wilk test to confirm suitability for parametric analysis. A repeated-measures analysis of variance (ANOVA) was applied to assess changes in VMD across different firing cycles, enabling within-group comparisons for each translucency type. Paired *t* -tests were used to detect any significant alterations in VMD due to repeated firings in pairwise comparisons. Independent *t* -tests were conducted to compare mean VMD values between the high- and low-translucency groups across all firing cycles. Statistical significance was set at *p* < 0.05.

## Results

**Fig. 3:**
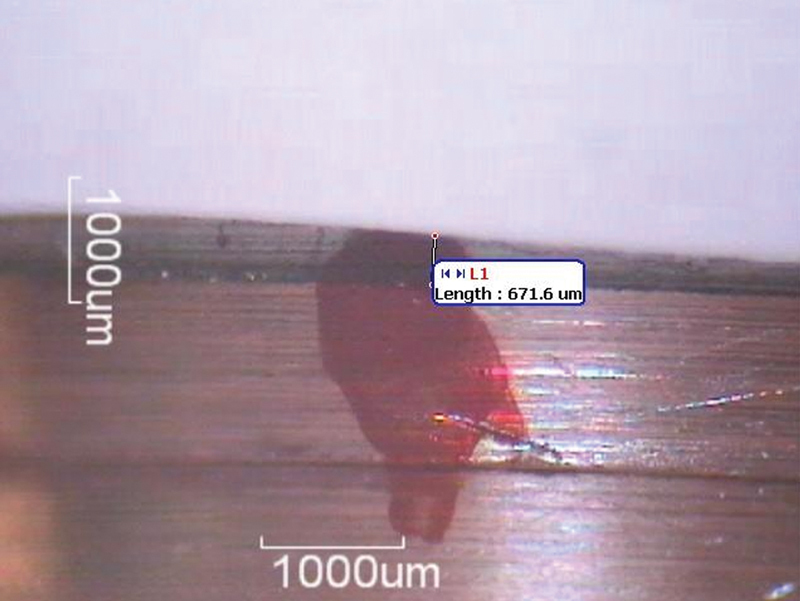
First marker of sample #3 of the low-translucency group in the 3rd firing cycle.

**Fig. 4:**
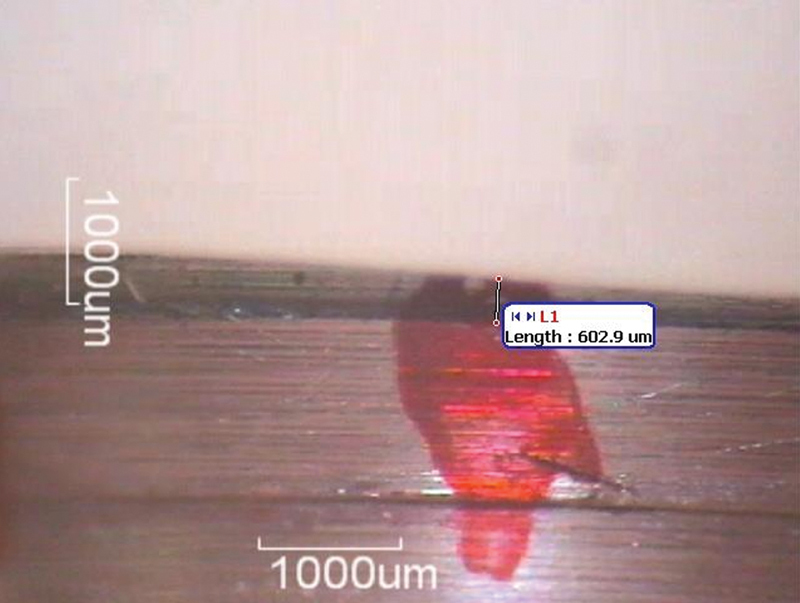
First marker of sample #4 of the high-translucency group in the 3rd firing cycle.

**Fig. 5:**
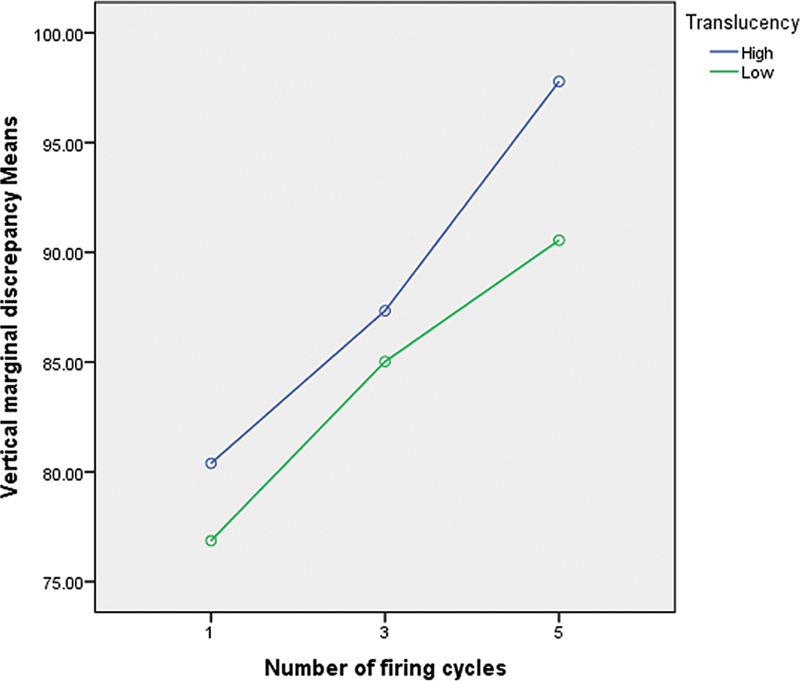
Mean vertical marginal discrepancy (VMD) of the samples in micrometers in high- and low-translucency groups according to the number of firing cycles.

**Fig. 6:**
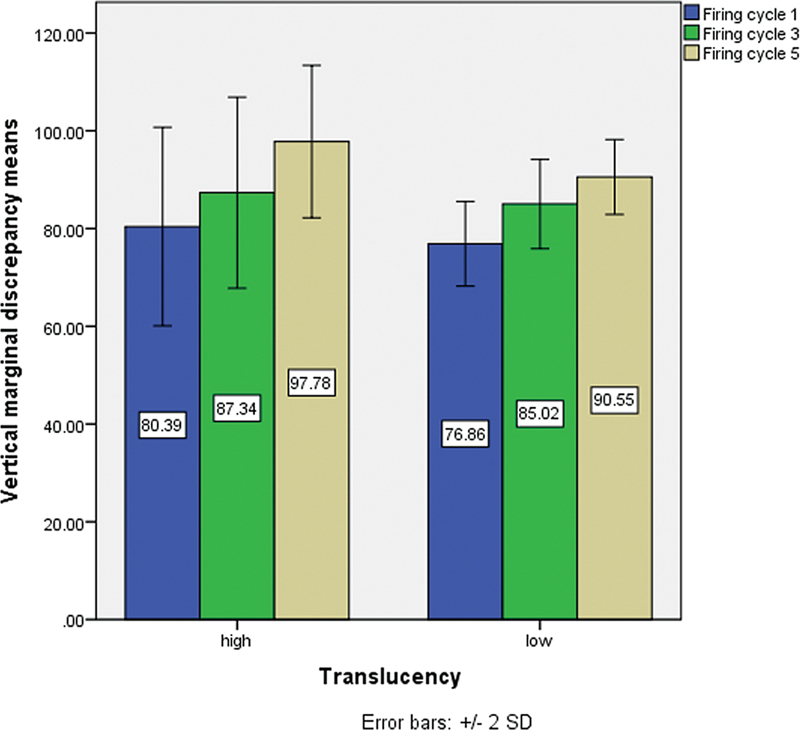
Mean vertical marginal discrepancy (VMD) of the samples in micrometers per firing cycle in high- and low-translucency groups.

A total of 240 measurements for VMD were taken at designated points on the abutment using an optical stereomicroscope ( Figs. 3 and 4 ). The mean VMD values were calculated at the eight points per sample and firing cycle. For each translucency group, the overall mean VMD across the eight points was calculated for each firing cycle ( Figs. 5 and 6 ).

The independent *t* -test for intergroup comparison revealed no significant differences in mean VMD between the high- and low-translucency groups across the three firing cycles (1, 3, and 5), with *p* -values of 0.496, 0.645, and 0.1, respectively ( Table 1 ).

**Table 1:** Mean and standard deviation (SD) of vertical marginal discrepancy (VMD) in micrometers for samples with high- and low-translucency, presented according to the number of firing cycles

Translucency	Firing cycle			*p* -Value b	Pairwise *p* -value c		
	1	3	5		1 vs. 3	3 vs. 5	1 vs. 5
High ( *N* = 5)	80.38 ± 10.15	87.33 ± 9.77	97.78 ± 7.80	< 0.001 d	< 0.001 d	< 0.001 d	< 0.001 d
Low ( *N* = 5)	76.86 ± 4.33	85.02 ± 4.56	90.55 ± 3.82	< 0.001 d	< 0.001 d	< 0.001 d	< 0.001 d
*p* -Value a	0.496	0.645	0.1				

Note: The table includes both intergroup (between high and low translucency) and intragroup (within each translucency group across firing cycles) comparisons.

a *p* -Value is the result of independent *t* -test.

b *p* -Value is as the result of repeated-measures analysis of variance (ANOVA).

c *p* -Value is as the result of paired *t* -test.

d Indicates a significant difference.

In intragroup comparison, when comparing the mean VMD across the three firing cycles irrespective of translucency, a repeated-measures ANOVA indicated a significant difference ( *p* < 0.001) between the cycles in both translucency groups. Further, paired *t* -tests showed that all pairwise comparisons between firing cycles were significant within each translucency group ( *p* < 0.001 for all), as detailed in Table 1 .

## Discussion

This study demonstrated that the VMD of monolithic zirconia crowns increased with repeated firing cycles. However, our study's findings emphasize the clinically acceptable VMD in high- and low-translucency monolithic zirconia crowns across multiple firing cycles (1, 3, and 5 times), with VMD levels consistently within acceptable ranges, irrespective of translucency levels. This finding suggests that translucency has a limited impact on changes in VMD during repeated firings, supporting the null hypothesis. This aligns with previous reports on zirconia crowns, where marginal gaps are commonly found between 10 and 160 µm, with clinically acceptable ranges identified as below 120 µm. 13 14 15 28

The fitness of a restoration to the prepared surface, both at the margin and internal surfaces, is crucial to achieving long-term clinical success. 29 Multiple approaches have been proposed to assess restoration fitness, including optical microscopy, transverse sectioning, 30 31 replica techniques, 32 profile projection, 33 and microtomography. 30 Among these, most studies evaluating the marginal fit of zirconia crowns utilize optical microscopy or replica techniques. In this study, we selected optical microscopy due to its nondestructive nature, ease of use, and practicality for measuring VMD. Optical microscopy has been validated for accuracy in zirconia crown assessment, confirming it as a reliable tool for marginal fit analysis. 34

Several factors contribute to variations in VMD values in all-ceramic crowns (zirconia or otherwise). Holmes et al defined marginal discrepancy as the vertical distance from the restoration margin to the margin of the prepared surface. 29 For ceramic crowns, a marginal gap between 50 and 120 µm is typically considered clinically acceptable, although gaps under 100 µm are more strongly associated with desirable durability. 11 12 In previous studies, the marginal gap of CAD/CAM-fabricated zirconia copings typically ranges between 10 and 160 μm, with most below 80 μm. 13 14 15 All-zirconia crowns specifically show even smaller gaps, often reported between 11 and 58 μm. 8 16

Although no consensus exists, Christensen reported that clinically acceptable subgingival marginal discrepancies range between 34 and 119 µm, with supragingival margins between 2 and 51 µm. 35 Lofstrom and Barkat's microscopic evaluations of clinically acceptable crowns reported marginal gaps from 7 to 65 µm, 36 while McLean and von Fraunhofer established an upper limit of 120 µm for clinical success. 28 However, using the finish line margin as a reference in measurements may introduce error due to rounding at the margin. 37 Although rounded custom abutments offer clinical relevance, shifting focal planes for measurement can reduce accuracy. 37

Cementation status also impacts marginal fit measurements, as the presence of luting material may obscure reference points. This study utilized a cement space setting of 30 μm in CAD/CAM software based on previous research indicating optimal adaptation at this level. 37 Samples were not cemented to maximize marginal fit accuracy, although this differs from clinical applications where cement type, viscosity, and technique affect fit. 38

Material thickness further affects VMD values. Zirconia thickness influences shrinkage during firing, affecting fit. 8 39 40 In this study, titanium abutments were used to prevent wear during preparation and measurement, following a 90-degree shoulder design suitable for zirconia crowns. 41 42 An occlusal convergence of 12% was applied, aligning with recommendations for optimal crown placement. 38 43 44

While these controlled variables provide insights into marginal fit, *in vitro* findings should be cautiously interpreted given the lack of dynamic oral environment conditions. Aging processes, including thermal and mechanical cycling, can influence VMD. Hung et al reported a negative effect of thermal cycling on fit, though Beschnidt and Strub noted no significant changes. 37 45 In this study, an aging process was not included; however, it is an area for future investigation to better simulate clinical conditions.

Measurement points are another critical factor. Groten et al recommended 20 to 25 points per crown for accurate marginal fit comparisons, although many studies use fewer points. In this study, we used eight points across five samples per translucency group to provide robust average VMD values. 46 47

Previous research on repeated firing cycles has mainly focused on nonzirconia ceramics, with limited data on monolithic zirconia. Balkaya et al's study on various ceramics demonstrated that repeated firings affect marginal fit, though glazing cycles do not. 24 Similarly, Cho et al found that repeated firings increase the marginal gap, with glazing slightly reducing it. 48 Vigolo and Fonzi, however, noted that in zirconia-based crowns, firing cycles did not significantly impact fit, suggesting that zirconia's unique properties may affect outcomes differently. 49

One limitation of this study is the relatively small sample size, which, although consistent with previous *in vitro* studies on zirconia restorations, may limit generalizability. To address this limitation, we increased the number of measurement points per crown to eight, providing a comprehensive assessment of VMD across regions. Nonetheless, larger-scale studies are warranted to validate these findings and enhance statistical power. Future research should consider broader sample sizes and more varied translucency levels and firing cycles. Additionally, exploring other factors, such as different cement types or aging conditions, would offer insights into the long-term clinical performance of zirconia crowns. Investigating aging effects through mechanical and thermal cycling could further simulate oral conditions, enhancing our understanding of zirconia's long-term stability. Finally, examining how variations in zirconia thickness and fabrication techniques impact marginal fit would elucidate other critical parameters influencing VMD.

## Conclusion

The findings of this study indicate that VMD increases with repeated firing cycles, with initially higher VMD values observed in the high-translucency group. However, no significant difference was found between high- and low-translucency groups in the VMD change across firing cycles, supporting the null hypothesis that translucency does not significantly impact VMD changes due to repeated firings. Regardless of translucency, repeated firings significantly affect VMD, increasing its value. Despite this, VMD values for both translucency groups remained below clinically acceptable limits, suggesting that monolithic zirconia crowns of both translucency types are suitable for use after repeated firings, provided case selection is appropriate for long-term success.
